# Entrance Examinations to Dental Colleges

**Published:** 1895-03

**Authors:** 


### ENTRANCE EXAMINATIONS TO DENTAL COLLEGES.

There are among dental teachers and writers men who lay much stress upon the entrance examinations for students, some putting it as high as a classical education. Indeed, in a recent editorial directed to the dental colleges in one of our contemporaries, such a plea was made.

This has always seemed to us a mistake. Of course, it is desirable that our classes should be composed of as good material as possible; the student should have at least a common-school education, and sufficient intelligence to comprehend the lectures and appreciate the value of his instructions and of time. There are men in every school who are not brilliant scholars, nor do they reach a very high mark in clinical work, though they may be earnest and persevering workers; but if their moral character be beyond reproach, they will fill their place in the activities of the world. In going into certain communities they will be appreciated, and possibly make a greater success of their-life work than some more scholarly man would in the same field.

The editor of the *Odontographic Journal* strikes the key-note of this subject when he says, Shall the standard be nailed in place by men of affairs, or by men as impracticable as learned tariff reformers, who read a little Latin very badly and less Greek worse?

Standards should be fixed by men of breadth, men capable of seeing that standards and standard-bearers imply standard supporters. One may recite languages either living or dead, likewise the contents of his Gray's Anatomy and other voluminous text-books, and yet fail utterly in his attempts at practical work. As Professors Peirce and Taft have expressed it, what we want is "a reasonable degree of scholarship." This means that men should not be kept in preparatory schools and colleges studying the fantastic courses which some of them present until the plastic stage is past.

If the entrance examinations should be such, as some, especially our English brethren, claim, as to keep out nearly every one but college-bred men, we should be sorry for the dental profession. Had this been the practice in years gone by, many of our very ablest teachers and representatives of to-day would not be found in the ranks. Far more important is it that more attention be given to the examinations throughout the course, especially the final one; not granting diplomas to men who it is known would be a discredit to their respective schools and to the profession.

It is unreasonable to expect our students, coming as many of them do from the rural districts, to be polished, scholarly men. True it is that there are among them men who are better adapted for other vocations; however, if they knock at the doors for admittance, our schools should not refuse them entrance (unless, of course, there is a good *moral* reason why they should not enter the profession), but should, by giving each one thorough, conscientious training, instruction, and care, endeavor to develop such men as will fill their places in the field of dentistry with credit.

The importance of moral worth or character of students has never, to our knowledge, had any bearing upon the entrance qualifications, and herein lies the secret of much discredit that has come upon the profession. It has frequently been asked, why dentists in many cities do not receive the same social recognition that is given representatives of other professions, and in many cases, if not all (if the truth were known), the answer would be, "Not from a lack of intellectuality, nor from want of skill as a dentist, but owing to the low moral fibre of the man."

Is it not time that the moral standard should be raised? The popular cry seems to be, "Raise the standard," but had we not better see to it that this embraces ethics and morals?

That there are throughout the land so many representatives of unsavory character is largely due to the lax conditions and requirements in this direction in our schools. Let no man hold a diploma from a reputable college unless he is an honorable, temperate, manly man.

